# Evaluation of the Performance of the IDvet IFN-Gamma Test for Diagnosis of Bovine Tuberculosis in Spain

**DOI:** 10.3389/fvets.2018.00229

**Published:** 2018-09-27

**Authors:** Maria Luisa de la Cruz, Adam J. Branscum, Jesus Nacar, Enrique Pages, Pilar Pozo, Andres Perez, Anna Grau, Jose Luis Saez, Lucia de Juan, Rosa Diaz, Olga Minguez, Julio Alvarez

**Affiliations:** ^1^VISAVET Health Surveillance Centre, Universidad Complutense de Madrid, Madrid, Spain; ^2^Biostatistics Program, Oregon State University, Corvallis, OR, United States; ^3^Dirección General de Producción Agropecuaria e Infraestructuras Agrarias, Consejería de Agricultura y Ganadería de la Junta de Castilla y León, Valladolid, Spain; ^4^Área de Ganadería, Dirección General de Medio Ambiente, Consejería de Medio Ambiente, Vivienda y Ordenación del Territorio de la Comunidad de Madrid, Madrid, Spain; ^5^MAEVA SERVET, S.L., Madrid, Spain; ^6^Department of Veterinary Population Medicine, College of Veterinary Medicine, University of Minnesota, Saint Paul, MN, United States; ^7^Ministerio de Agricultura y Pesca, Alimentación y Medio Ambiente, Madrid, Spain; ^8^Departamento de Sanidad Animal, Facultad de Veterinaria, Universidad Complutense de Madrid, Madrid, Spain

**Keywords:** bovine tuberculosis, cattle, diagnosis, interferon-gamma, bayesian modeling

## Abstract

In Spain, the national bovine tuberculosis (bTB) eradication program is based on yearly skin testing of every ≥6 weeks old animal using the single or comparative tuberculin test and parallel use of the interferon-gamma (IFN-γ) assay as an ancillary diagnostic test in infected herds. There are several versions of the latter. Recently, a new commercial IDvet IFN-γ assay has been authorized for use in the program, but there is limited scientific evidence about its performance in different epidemiological settings. Therefore, two studies to evaluate the performance of the IDvet assay were conducted. In study 1, a concordance analysis between the new IDvet and the Bovigam IFN-γ assay in use in Spain for over 10 years was conducted. In study 2, results from the IDvet assay when applied in tandem with a single intradermal tuberculin (SIT) test were used to evaluate the concordance between both tests and to estimate their sensitivity (Se) and specificity (Sp) using a Bayesian latent-class model. Field data from cattle herds located in Madrid and Castilla y Leon (Spain) were collected. For study 1, herd selection was based on a high expected prevalence of reactors to the IFN-γ assay, while herds were selected at random to estimate Se and Sp of the new IDvet assay in study 2. Agreement between the results obtained with both kits for IFN-γ assay was poor (Kappa = 0.20), and a receiver operating characteristic (ROC) analysis indicated a low Se of the new IDvet relative to the Bovigam in a heavily bTB infected population. The Bayesian latent-class analysis estimated the Se of the IDvet assay to be 36.7% [95% probability posterior interval (PPI) 14.7–78.8%] with estimated Sp close to 100% when the cut-off recommended by the manufacturer (35) was applied. At the alternative cut-off values of 16 and 4, the estimated Se of the IDvet assay increased to 49.0% (PPI: 24.8–94.1%) and 56.0% (PPI: 30.8–96.3%), respectively, while maintaining a high specificity. The results suggest that the new IDvet assay may have lower sensitivity than the Bovigam for diagnosis of bTB in cattle herds in Spain, and that adjusting its cut-off might be considered.

## Introduction

Bovine tuberculosis (bTB), caused by members of the *Mycobacterium tuberculosis* complex (mainly *M. bovis*, and to a lesser extent, *M. caprae*) is an important zoonotic disease with a global distribution that has major implications for both animal and human health (1). Implementation of control and eradication programs has led to a significant decrease of the bTB prevalence and to disease eradication in many industrialized countries (1–3). However, eradication efforts have not been uniformly successful, in part because currently available diagnostic tests cannot correctly determine the *M. bovis* infection status of all tested cattle (4). *M. bovis* infection in cattle is usually chronic, can remain subclinical for a long period, and infected cattle can become infectious long before they exhibit clinical signs of bTB (4). When present, the clinical signs of bTB are not pathognomonic. As a result, control strategies have been based on early detection and removal of infected animals from a herd by applying ante-mortem diagnostic tests and routine post-mortem surveillance in abattoir. Hence, determining the accuracy of diagnostic tests for bTB is of paramount importance (5).

Diagnostic tests used for detection of bTB in cattle are mainly based on detecting the cellular mediated immune (CMI) response, which is triggered in the early stages of infection (6). A popular diagnostic technique for this purpose, the single intradermal tuberculin (SIT) test, is based on the inoculation of the bovine purified protein derivative (PPD) in the skin of the neck or in the caudal fold (7). In certain settings the response to the avian PPD inoculated in the other side of the neck is also measured and compared with the bovine response in what is known as the single intradermal comparative cervical tuberculin (SICCT) test, used as the routine screening test in countries as the UK and Ireland. In the last 25 years, an additional diagnostic tool for measuring the CMI response, the interferon-gamma (IFN-γ) release assay, has been increasingly used (8). This test is based on the detection of IFN-γ produced by lymphocytes present in blood samples stimulated with specific antigens (typically bovine and avian PPD); blood samples are then centrifuged after the stimulation and the resulting plasma is analyzed using a sandwich ELISA (8). As an ancillary test to skin test, the IFN-γ assay has led to increased sensitivity when used in infected herds (9), in part because it can identify animals in an earlier infection stage than the SIT test (10). Therefore, in the European Union its ancillary use is recommended in infected herds located in areas with endemic bTB (annex B of Council Directive 64/432/EEC), such as certain areas of Spain (11).

The first commercially available IFN-γ test (Bovigam, Thermo Fisher Scientific, Waltham, MA, USA) (12) has been used in multiple European countries, including Spain, and its performance has been extensively evaluated under field conditions (4, 9, 13–19). Recently, another version of the IFN-γ assay has become commercially available (ID Screen® Ruminant IFN-γ, IDvet, Grabels, France), but due to its recent development there is limited information available about its performance in different epidemiological settings.

The assessment of test accuracy (i.e., sensitivity, Se, and specificity, Sp) is challenging when infection status cannot be determined because, for instance, a perfect reference test does not exist or is too invasive for widespread use, as is the case for bTB, in which the usual reference test, isolation of the causative agent, is costly, slow and has a very limited sensitivity in a large proportion of the infected animals (4). Latent class analysis is a modern approach to estimating the accuracy of diagnostic tests in the absence of a perfect reference test (20, 21). In Bayesian latent class analysis, parameters (e.g., sensitivities, specificities, and prevalences) can be estimated by combining prior knowledge with information from the current data. Bayesian latent class models have been used to estimate the diagnostic performance of bTB tests (5, 22–27), often revealing important disagreements between the prior knowledge and the study data.

In Spain, a national bTB eradication program is in place since the 80's, and since 2006 contemplated the addition of the IFN-γ test as an ancillary test to increase diagnostic sensitivity according to the European and Spanish regulations. During this period the bTB herd prevalence has decreased from >10 to 2.87% in 2016, but the progress in the last decade has been more limited (11). Here, we conducted a study to evaluate the performance of the new commercial IFN-γ test (called thereafter IDvet test) that has been recently authorized for use as part of the Spanish bTB eradication program. Field data were collected to accomplish two goals, namely to conduct a concordance analysis between the new IDvet test and the Bovigam IFN-γ assay (called thereafter Bovigam test), which has been used in Spain for over 10 years (study 1) and to assess the concordance between the IDvet and SIT tests and estimate their Se and Sp in the absence of a gold standard (study 2).

## Materials and methods

### Study population

#### Study 1. evaluate the IDvet test using the bovigam test as a reference

The new IFN-γ assay (ID Screen® Ruminant IFN-g, IDvet, Grabels, France) and the pre-existing IFN-γ test (Bovigam®, Thermo Fisher Scientific, Waltham, MA, USA) were compared using results from 1,181 cattle from 18 herds located in the regions of Madrid [884 cattle (74.9%) from 11 herds] and Castilla y Leon [297 cattle (25.1%) from 7 herds], in central and west-central Spain, respectively. These herds were selected from among those being tested using the IFN-γ assay during the bTB eradication program in 2015 and 2016 based on a high expected prevalence of reactors to the IFN-γ assay. A detailed explanation on the epidemiological situations in which the test is implemented in Spain is available elsewhere (28). The majority of the sample (84.1% of the animals and 88.9% of the herds) was represented by beef herds (993 animals and 16 herds), followed by bullfighting (103 animals and one herd) and dairy herds (85 animals and one herd).

#### Study 2. estimate the Se and Sp of the IDvet test in the field

Test results were obtained from 8,426 cattle (78 herds) subjected to the SIT test and IDvet test during 2016. Herds were again located in the Madrid [22 herds, 1,550 animals (18.4%)] and Castilla y Leon [56 herds, 6,876 animals (81.6%)] regions, and they were randomly selected among the bTB confirmed infected herds in the two regions in 2016 in which the IFN-γ assay had been implemented according to the Spanish eradication program (11). Beef was again the predominant production type (19 herds and 1,204 animals in Madrid; 41 herds and 4,382 animals in Castilla y Leon), followed by dairy (one herd-44 animals and 10 herds-1676 animals in Madrid and Castilla y Leon, respectively), bullfighting (two herds-300 animals and two herds-339 animals in Madrid and Castilla y Leon, respectively), and mixed farms (3 herds and 481 animals, all in Castilla y Leon).

### Diagnostic tests

#### Single cervical intradermal tuberculin test (SIT)

The SIT test was performed according to European and Spanish regulations (RD2611/1996, transposition of annex A of Council Directive 64/432/EEC) by field practitioners in all >6 week-old animals at the herd by intradermal inoculation of 0.1 ml of the official bovine PPD (CZ Veterinaria, Porriño, Spain) in the anterior neck area (29). After 72 h, animals with a >2 mm increase of the skin fold thickness (or with presence of clinical signs at the inoculation site) were considered reactors (severe interpretation) following the Spanish National Bovine Tuberculosis Eradication Program (11) and culled within 15 days.

#### IFN-γ assay

Heparinized blood samples were collected from every animal prior to intradermal injection of the PPDs, and delivered to the laboratory in Madrid or Castilla y Leon within 8 h of collection at room temperature, according to the Spanish National Bovine Tuberculosis Eradication Program (11). Stimulation with bovine and avian PPDs (CZ Veterinaria) at a final concentration of 20 μg/ml, and nil antigen phosphate buffer saline (PBS) was carried out as described elsewhere (8). Plasma samples were harvested after centrifugation and stored at−20°C until testing for detection of the IFN-γ with one or both sandwich ELISA evaluated here.

##### Bovigam® IFN-γ

The Bovigam test was carried out following procedures described elsewhere (12). An animal was considered positive when the optical density (OD) of the aliquot stimulated with bovine PPD minus the OD of the nil (bovine IFN) was ≥0.05 and greater than the OD of the sample stimulated with avian PPD minus the nil (avian IFN), and negative in any other case (11).

##### ID screen® ruminant IFN- γ, IDvet

. The IDvet test was performed according to the manufacturer instructions (IFNG ver 0617 ES). Briefly, samples were divided into three aliquots and incubated with PBS (blank), bovine (activated sample) or avian (control sample) PPD. When OD values>2.5 were obtained in the blank or both the control and activated sample (suggestive of unspecific reactions) samples were diluted 1:5 in order to bring OD levels into the linear region of OD measurement and reanalyzed as indicated by the manufacturer. Results were then transformed into sample-to-positive ratios (S/P):

$$\begin{array}{l} \frac{S}{P}=\left(\frac{OD\;activated\;sample-OD\;control\;sample}{\begin{array}{c} OD\;mean\;kit\;positive\;control-OD\;meankit\\ negative\;control \end{array}}\right)*100 \end{array}$$

Samples were considered positive when the S/P ratio was ≥35 according to the manufacturer instructions. In addition, alternative cut-off points were evaluated, as described below.

### Statistical analysis

#### Concordance analysis

Agreement between the qualitative results obtained from both IFN-γ kits (study 1) and between the IDvet test and the SIT test (study 2) was measured using the kappa statistic. Receiver operating characteristic (ROC) curves were used to evaluate the performance of the IDvet test at different cut-offs in relation to the Bovigam (study 1), with Youden's index used to assess optimal cut-off values. The same tests were performed using only the data from beef cattle. These analyses were carried out using SPSS V. 20 (IBM Inc., Chicago, IL, USA) and the “pROC” (30) and “ROCR” (31) packages from in R 3.4 (32).

#### Latent class analysis

We followed the guidelines for reporting of diagnostic accuracy in studies that use Bayesian latent class models (STARD-BLCM) (21).

A Bayesian latent-class model was used to estimate the Se and Sp of the SIT test and the IDvet test in infected herds (study 2), in the absence of a gold standard (33, 34). Samples were considered to belong to two different populations based on the region of origin (Madrid and Castilla y Leon) of herds and the two tests were assumed to be conditionally dependent (33) since both are based on the detection of the cell-mediated immune response (10).

Prior beta distributions for the Se and Sp of the SIT and IDvet tests were built according to reported/estimated values (5, 7, 35–38) (Table 1) based on the most likely value and a low 95% credibility interval using ParameterSolver V3.0 (University of Texas MD Anderson Cancer Center). For the IDvet test, larger prior standard deviations were used to reflect the uncertainty in its Se and Sp due to the lack of prior information for this kit and the findings from study 1 (see Results). Covariances between the SIT test and IDvet test for infected and non-infected subpopulations were specified as previously described (39), and these parameters were modeled with uniform prior distributions related to the Se and Sp of the tests (40) (Supplementary File 1).

**Table 1:** Prior estimates of sensitivity and specificity of the SIT test and the IDvet test.

**Diagnostic test**	**Performance measure**	**Prior estimates**		**Reference**	
		**Mode and 5th percentile**	**Beta distribution**	**Authors, year**	**Reported/estimated values**
SIT test	Sensitivity (%)	69 (>40)	alpha: 5.65 beta: 2.71	Alvarez et al. (5)	69.4 (40.1–92.2)
				de la Rua-Domenech et al. (4)	83.9 (63.2–100)
				Monaghan et al. (7)	68–95
				Wood et al. (37)	68.1
				Wood et al. (36)	65.6 (56.6–73.9)
	Specificity (%)	95 (>75)	alpha: 8.65 beta: 0.73	Alvarez et al. (5)	99.4 (98.7–99.9)
				de la Rua-Domenech et al. (4)	96.8 (75.5–99.0)
				Monaghan et al. (7)	96–99
				Wood et al. (37)	96.7
IDvet test	Sensitivity (%)	90 (>50)	alpha: 3.35 beta: 0.62	Alvarez et al. (5)	89.3 (77.5–97.2)
				de la Rua-Domenech et al. (4)	87.6 (73.0–100)
				Gormley et al. (35)	88
				Nunez-Garcia et al. (38)	67 (49–82)
				Wood et al. (37)	81.8
				Wood et al. (36)	80.8 (72.8–87.3)
	Specificity (%)	90 (>80)	alpha: 33.1 beta: 3.97	Alvarez et al. (5)	85.7 (84.4–87.6)
				de la Rua-Domenech et al. (4)	96.6 (85.0–99.6)
				Gormley et al. (35)	95
				Nunez-Garcia et al. (38)	98 (96–99)
				Wood et al. (37)	99.1
				Wood et al. (36)	90

Based on data collected in 2014-2015 from infected herds in Castilla y Leon and Madrid, the common prior distribution for the bTB prevalence was assigned a mode of 5% and a 95th percentile of 20% [specifically, beta(0.99, 13.4)] for both regions.

A sensitivity analysis to evaluate the impact of the priors on the results of the model was conducted using diffuse uniform (0, 1) distributions alternatively for the Se and Sp of each test. Model estimates (posterior medians and 95% posterior probability intervals, PPI) were compared with those obtained using the informative priors. Models were also run only using the data from beef cattle as an additional sensitivity analysis. In addition, alternative cut-offs for the IDvet test (S/P ratio = 16 and 4) were evaluated.

Three Markov chain Monte Carlo runs were implemented in order to visually assess convergence and mixing of the chains. Convergence was also assessed using the Gelman-Rubin diagnostic (41). Posterior inference was based on 5,000 iterations after discarding the first 2,500 as burn-in. Autocorrelation was eliminated through thinning the chains by collecting one in 10 consecutive samples. All analyses were conducted using OpenBUGS V. 3.2.3 (42) called through R, version 3.4 (32) using the “R2OpenBUGS” package (43). The OpenBUGS code is provided as supplementary material (Supplementary File 1).

## Results

### Concordance analysis

**Figure 1:**
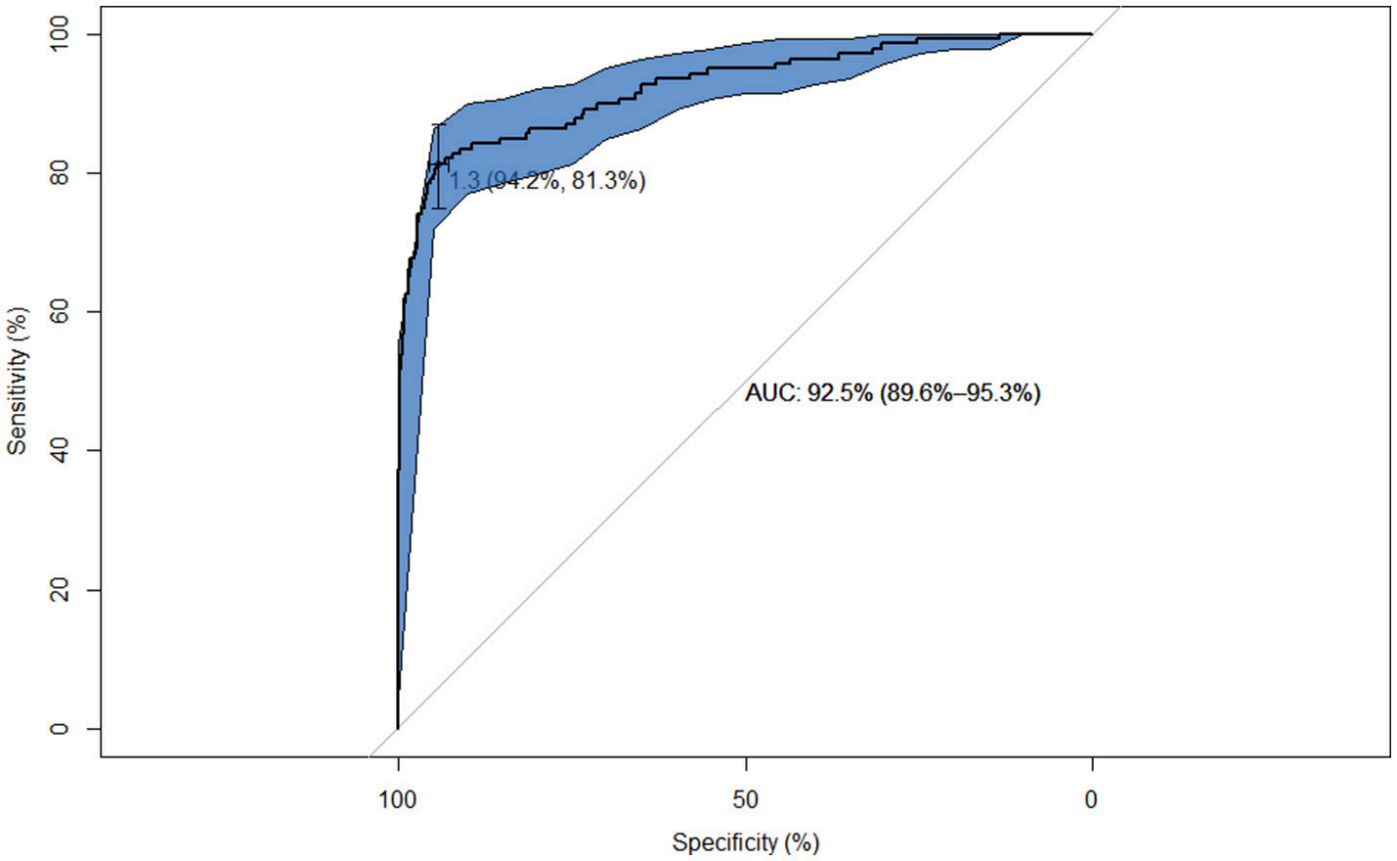
ROC curve of the performance of the IDvet test relative to the Bovigam test on 1,181 samples from animals analyzed with both tests. The cross points the location for the optimal cut-off for maximization of the Youden's index for the IDvet test (1.3) along with the Sp and Se at this cut-off.

The number of reactors to each test in both studies are shown in Table 2. Agreement between the results obtained with the Bovigam and the IDvet tests (study 1) was poor (Kappa = 0.20), with most of the discordant results being positive in the former and negative in the new test (Table 2). The ROC analysis indicated that at the manufacturer recommended cut-off of 35 for the IDvet, Se relative to the Bovigam test was low (Se = 15.1% and Sp = 99.9%). Better agreement was achieved when lower cut-offs (16 and 4) were used (Kappa = 0.52 and 0.71, with Se = 38.8 and 65.5, respectively, and Sp > 98.5%). A high value of 92.5% (95% CI: 89.6–95.3%) was obtained for the AUC (Figure 1). The optimal cut-off point according to Youden's index in relation to the Bovigam was 1.3 (yielding Se = 81.3% and Sp = 94.2%). When only beef cattle were considered, very similar Kappa values were obtained regardless of the cut-off used (0.20, 0.52, and 0.70 for cut-offs 35, 16, and 4, respectively), with also a very high AUC (90.9%) and a similar optimal cut-off point (1.7).

**Table 2:** Number of reactors to the IDvet test and to the Bovigam test performed on 1,181 cattle (study 1), and number of reactors to the IDvet test and to the SIT test (severe interpretation) performed on 8,426 cattle (study 2) from Madrid and Castilla y Leon (Spain).

				**IDvet test (35)**		**Total**	
				**Negatives**	**Positives**		
Study 1		Bovigam test (0.05)	Negatives	1,087	1	1,181	
			Positives	82	11		
Study 2	Madrid	SIT test (severe int)	Negatives	1,509	8	1,548	8,426
			Positives	22	9		
	Castilla y Leon	SIT test (severe int)	Negatives	6,738	76	6,878	
			Positives	35	29		

Agreement between the IDvet test and the SIT test (study 2) was slightly higher (Kappa = 0.34), although important differences were still observed (Table 2). When the results were analyzed separately by production type, the agreement was higher for beef cattle (Kappa = 0.43) and close to zero for dairy and mixed herds (Kappa = 0.08 and −0.01, respectively), whereas it could not be calculated for bullfighting cattle due to the lack of SIT reactors. For cut-off values of 16 and 4, the agreement between tests decreased to Kappa values of 0.27 and 0.14, respectively (Supplementary Table 1).

### Latent class analysis

The Bayesian latent class analysis yielded posterior estimates (median and 95% PPI) for the IDvet test of Se = 36.7% (14.7–78.8%), with very high Sp (Table 3). The posterior distribution of Se for the IDvet test was shifted below its prior distribution (Supplementary Figure 1), whereas the opposite was true for its Sp. In contrast, the posterior estimates of the performance of the SIT test were largely in agreement with the prior information.

**Table 3:** Posterior estimates (median and 95% posterior probability interval) for sensitivity, specificity and the mean of the prevalence distribution (%) obtained for the combination of IDvet test and SIT test on 8,426 cattle from Madrid and Castilla y Leon (Spain), for different prior distributions and IDvet alternative cut-off points.

**Model**	**Priors**	**Diagnostic test**	**Sensitivity**	**Specificity**	**Prevalence**	
					**Madrid**	**Castilla y leon**
Original	Original priors (Table 1)	SIT test	78.68 (49.28–95.00)	99.53 (98.95–99.98)	1.85 (0.51–3.34)	0.64 (0.04–1.43)
		IDvet 35^a^	36.69 (14.66–78.81)	98.78 (98.41–99.18)		
Alternative cut-off points for IDvet	Original priors (Table 1)	SIT test	76.59 (47.06–94.51)	99.55 (98.95–99.99)	1.90 (0.59–3.50)	0.69 (0.04–1.56)
		IDvet 16^b^	49.03 (24.85–94.13)	97.86 (97.38–98.36)		
	Original priors (Table 1)	SIT test	76.41 (46.11–94.41)	99.61 (98.99–99.99)	1.96 (0.70–3.62)	0.78 (0.06–1.71)
		IDvet 4^c^	55.98 (30.76–96.34)	93.89 (93.25–94.57)		

a *Cut-off recommended by the manufacturer*.

b *Cut-off for interpretation = 16*.

c *Cut-off for interpretation = 4*.

Conditional dependence between the SIT test and the IDvet test was estimated to be very low in both the infected (correlation coefficient of positive results: −0.002, 95% PPI −0.13 to 0.09) and non-infected (correlation coefficient of negative results: 0.002, 95% PPI 0.00 to 0.004) populations, suggesting a possible conditional independence between the results of each test. Note that, in general, conditional independence occurs when Sp's are close to 100%.

A sensitivity analysis (Supplementary Table 2) resulted in no major changes to the posterior distribution of Se and Sp for both tests (the magnitudes of all percent differences were <8%), except when a uniform prior was used for the Se of the IDvet test assay where a percent decrease of 41% was observed for the posterior median (36.7 vs. 21.6). Prevalence estimates were similar across analyses that used different priors (e.g., overlapping 95% PPI's), and were 2–3 times higher in the region of Madrid than in Castilla y Leon. When only values from beef herds were used in the analysis, the 95% PPI for the sensitivity of the IDvet test was similar (19–88%) but a higher median value was found (60.9%), while changes in the posterior estimates for the sensitivity of the SIT test and the specificities of both techniques were small (12% of median estimates). Similar estimates were obtained when using a burn-in of 20,000 posterior iterates and 50,000 total iterates.

At cut-off points of 16 and 4 for the interpretation of the IDvet test, the estimated Se of the IFN-γ assay increased (as expected) to 49.0 and 56.0%, respectively, while maintaining a high specificity (Table 3). The posterior estimates for the performance of the SIT test and the bTB prevalence were not affected by the cut-off applied in the IDvet test (Table 3).

There was no evidence for lack of convergence of the Markov chains used to simulate from the posterior distribution (Supplementary Figure 2), as indicated by graphical assessment of the chains and the Gelman-Rubin statistic < 1.001 for all parameters.

## Discussion

Multiple factors related with the host, the pathogen and the environment may affect the performance of a diagnostic test, and, therefore, extrapolation of results obtained in different epidemiological settings may lead to biased and misleading conclusions. For this reason, in the studies presented here we aimed at estimating, by using a variety of analytical approaches, the performance of the new commercial IDvet test under field conditions in bTB-infected herds of Spain. Even though both Bovigam and IDvet tests share the same target (IFN-γ produced by lymphocytes stimulated with bovine PPD), when the results obtained in both assays were compared (study 1), the agreement was poor (Kappa = 0.20). In addition, the Se of the IDvet test relative to the Bovigam test in a population formed by heavily bTB infected cattle herds was very low (15.1%), although given the limited specificity of the Bovigam (with estimates between 84.4 and 99.6%) (4, 5, 35–38) a proportion of false positive reactions to this test could be expected, and therefore this figure could be an underestimation of its true sensitivity. Nevertheless, the high AUC values obtained when the quantitative readings obtained in the IDvet test were compared with the qualitative response in the Bovigam test (92.5, 90.9% when only beef animals were considered) suggested that there was in fact a close relationship between the response measured in both tests. Moreover, the ROC analysis suggested that decreasing the cut-off in the IDvet test (thus requiring a lower difference between the response recorded in the sample stimulated with bovine PPD compared with the avian PPD to define an animal as a reactor) could lead to a substantial increase in the agreement between both tests (Figure 1), despite the different calculations used to define the positive status (see Material and Methods). In addition to the comparison of the responses after the *in-vitro* stimulation with avian and bovine PPD to define a reactor considered in both the Bovigam and IDvet tests, the protocol of the IDvet test includes an extra step (dilution of samples) to avoid false positive reactions due to sensitization with other cross-reacting microorganisms such as *Mycobacterium avium* subsp. *paratuberculosis*, that are known to affect the performance of IFN-γ based assays in domestic ruminants (44). Although this could have theoretically contributed to the observed differences between both tests, less than 1% of the samples included in study 1 had to be diluted because of high readings in the control and activated samples, and therefore this was not a major source of variation in our study.

A limited agreement between the IDvet test and the SIT test (Kappa = 0.34; study 2) was also observed. This is consistent with a field scenario in which animals are subjected to frequent skin testing and SIT-reactors have been already removed when the IFN-γ assay is introduced for the first time in infected herds (7, 10, 12, 45–47). This limited agreement between the SIT test and the IDvet test, coupled with the low posterior estimates for the codependence terms obtained in the latent class analysis (correlation coefficient range: −0.13 to 0.09 and 0.00 to 0.004, for infected and non-infected animals, respectively) are in agreement with estimates obtained for the Bovigam test (5), and reinforce the potential usefulness of the application of IFN-γ based assays in parallel to the SIT test to maximize the diagnostic sensitivity (45, 48, 49). In the case of the IDvet test, however, results from the Bayesian latent class model (study 2) confirmed the apparent lower Se of the IDvet test compared with the Bovigam test observed in study 1, since posterior estimates of the IDvet Se were significantly lower than those recently estimated for the Bovigam test using a similar—though wider—prior (36.7, 95% PPI 14.7–78.8, vs. 89.3, 95% PPI 77.5–97.2 estimated previously for the Bovigam test) (5). The very large uncertainty in the IDvet Se posterior estimates may be also influenced by the small number of IDvet reactors in the sample (Table 2). In contrast, the results obtained for the SIT test in this study and the previous one were comparable, with higher median estimates obtained here but very similar PPI (78.7, 95% PPI 49.3–95.0, compared with 69.4, 95% PPI 40.1–92.2) (5). When only data from beef herds were analyzed, a higher posterior median value for Se of the IDvet was obtained (60% compared with 37% when analyzing all animals) although the 95% PPI was similar, and values were nevertheless still lower than previous estimates obtained for the Bovigam test as well as the priors used in the analysis. In fact, the sensitivity analysis revealed a conflict between the prior information used for the Se of the IDvet test in this study and the data, because when a non-informative prior was used the posterior estimates for its Se were even lower compared to the use of the informative prior using all animals (Supplementary Table 2) or only beef cattle (data not shown). This confirms the hypothesis that the IDvet test had a significantly lower Se compared to the early test (Table 3).

Our results contradict those obtained in experimental studies carried out in France, Belgium and Mexico, in which high values of Se (88.3, 95% CI: 81.1–95.5) and Sp (99.0, 95% CI: 98.4–99.6) were reported (50). Those trials however involved a relatively limited number of animals (*n* = 77) already positive to either PCR, culture or the SIT test, thus representing a potentially biased subpopulation of all infected animals present in an infected herd, what could lead to an overestimation of the sensitivity of the test (51).

Similar to the observation of study 1, results from study 2 suggest that a decrease in the cut-off value in the interpretation of the IDvet test could substantially increase the sensitivity of the test (see Table 3 and Figure 1), in agreement with a previous study (47), while maintaining a high specificity (median posterior values >93.9%). Given that in the EU the Bovigam test is applied to maximize the number of infected animals detected, this may be a reasonable approach when samples from infected herds are analyzed. However, further validation of this hypothesis may be required.

Here, a two-population approach was used because infected herds located in the same region were expected to present similar prevalence levels, as reflected in the official bTB reports for the previous years (52). In fact, the ratio between the estimated bTB prevalence in each region (Madrid/Castilla y Leon; Table 3) and the reported in 2016 is similar, 2.9 and 2.6, respectively, even though posterior estimates were below the most likely prior values.

The use of a latent class analysis allowed overcoming the limitations of the gold-standard approach, since all available reference tests for bovine tuberculosis have low sensitivity particularly in early stages of infection, when its detection is most critical (4, 53). However, for the comparison between the performance of the IDvet and Bovigam tests a latent class model was not used because the population had been selected based on an expected high prevalence of infection. Hence, the assessed population was not representative of the field situation, so that it only allowed a comparison of the performance of the tests in that very specific context. Still, results obtained in that potentially biased population suggested that the IDvet test could have a lower Se compared with the Bovigam test. For study 2, animals were selected randomly from infected herds in which the SIT test and the IDvet were being implemented routinely, and hence were considered truly representative of the situation in which the performance of the test was intended to be determined.

In conclusion, our results suggest that the IDvet test may have a lower sensitivity than the Bovigam for diagnosis of bTB in cattle herds in Spain when the cut-off recommended by the manufacturer is applied. Decreasing the cut-off may result in a substantial increase of the sensitivity while maintaining a high specificity, although generalization of that result would require verification under alternative epidemiological settings and conditions.

## Ethics statement

Animals included in this study were only subjected to the regular tests performed in the framework of the Spanish official program for eradication of bovine tuberculosis, and therefore no experimental research on animals was conducted in this study.

## Author contributions

MdC, AB, PP, and JA conceived and performed the statistical analyses. MdC, and JA drafted the manuscript. JN, EP, AG, JS, RD, and OM participated in the generation, collection and curation of the data, and collaborated in interpretation of the results. AP, LdJ, and JA designed the study and coordinated the work. All authors revised critically the manuscript. All authors read and approved the final manuscript.

### Conflict of interest statement

The authors declare that the research was conducted in the absence of any commercial or financial relationships that could be construed as a potential conflict of interest.
