# Supplementary material for: Evaluation of the Performance of the IDvet IFN-Gamma Test for Diagnosis of Bovine Tuberculosis in Spain
Source: Front Vet Sci. 2018 Sep 27;5:229. doi: 10.3389/fvets.2018.00229 (PMC6171474; doi:10.3389/fvets.2018.00229)
Supplement: Supplementary file 3 [file Data_Sheet_1.DOCX]

model{

y1[1:4] ~ dmulti(p[1:4], n1)

y2[1:4] ~ dmulti(p[5:8], n2)

p[1] <- pi1*((1-Se1)*(1-Se2)+covDp) + (1-pi1)*(Sp1*Sp2+covDn)

p[2] <- pi1*(Se1*(1-Se2)-covDp) + (1-pi1)*((1-Sp1)*Sp2-covDn)

p[3] <- pi1*((1-Se1)*Se2-covDp) + (1-pi1)*(Sp1*(1-Sp2)-covDn)

p[4] <- pi1*(Se1*Se2+covDp) + (1-pi1)*((1-Sp1)*(1-Sp2)+covDn)

p[5] <- pi2*((1-Se1)*(1-Se2)+covDp) + (1-pi2)*(Sp1*Sp2+covDn)

p[6] <- pi2*(Se1*(1-Se2)-covDp) + (1-pi2)*((1-Sp1)*Sp2-covDn)

p[7] <- pi2*((1-Se1)*Se2-covDp) + (1-pi2)*(Sp1*(1-Sp2)-covDn)

p[8] <- pi2*(Se1*Se2+covDp) + (1-pi2)*((1-Sp1)*(1-Sp2)+covDn)

pi1 ~ dbeta(0.99131905,13.353895)

pi2 ~ dbeta(0.99131905,13.353895)

Se1 ~ dbeta(5.647922,2.7137574)

Sp1 ~ dbeta(8.6472167,0.73148716)

Se2 ~ dbeta(3.3538309,0.62062971)

Sp2 ~ dbeta(33.120369,3.9707257)

ls <- (Se1-1)*(1-Se2)

us <- min(Se1,Se2) - Se1*Se2

lc <- (Sp1-1)*(1-Sp2)

uc <- min(Sp1,Sp2) - Sp1*Sp2

covDn ~ dunif(lc, uc)

covDp ~ dunif(ls, us)

rhoDp <- covDp / sqrt(Se1*(1-Se1)*Se2*(1-Se2))

rhoDn <- covDn / sqrt(Sp1*(1-Sp1)*Sp2*(1-Sp2))

}
