# Supplementary material for: Evaluation of the Performance of the IDvet IFN-Gamma Test for Diagnosis of Bovine Tuberculosis in Spain
Source: Front Vet Sci. 2018 Sep 27;5:229. doi: 10.3389/fvets.2018.00229 (PMC6171474; doi:10.3389/fvets.2018.00229)

Supplementary Figure 1. Prior (dashed) and posterior (continuous) distributions for the sensitivity of the SIT test (grey) and the IDvet IFN-γ assay (black).

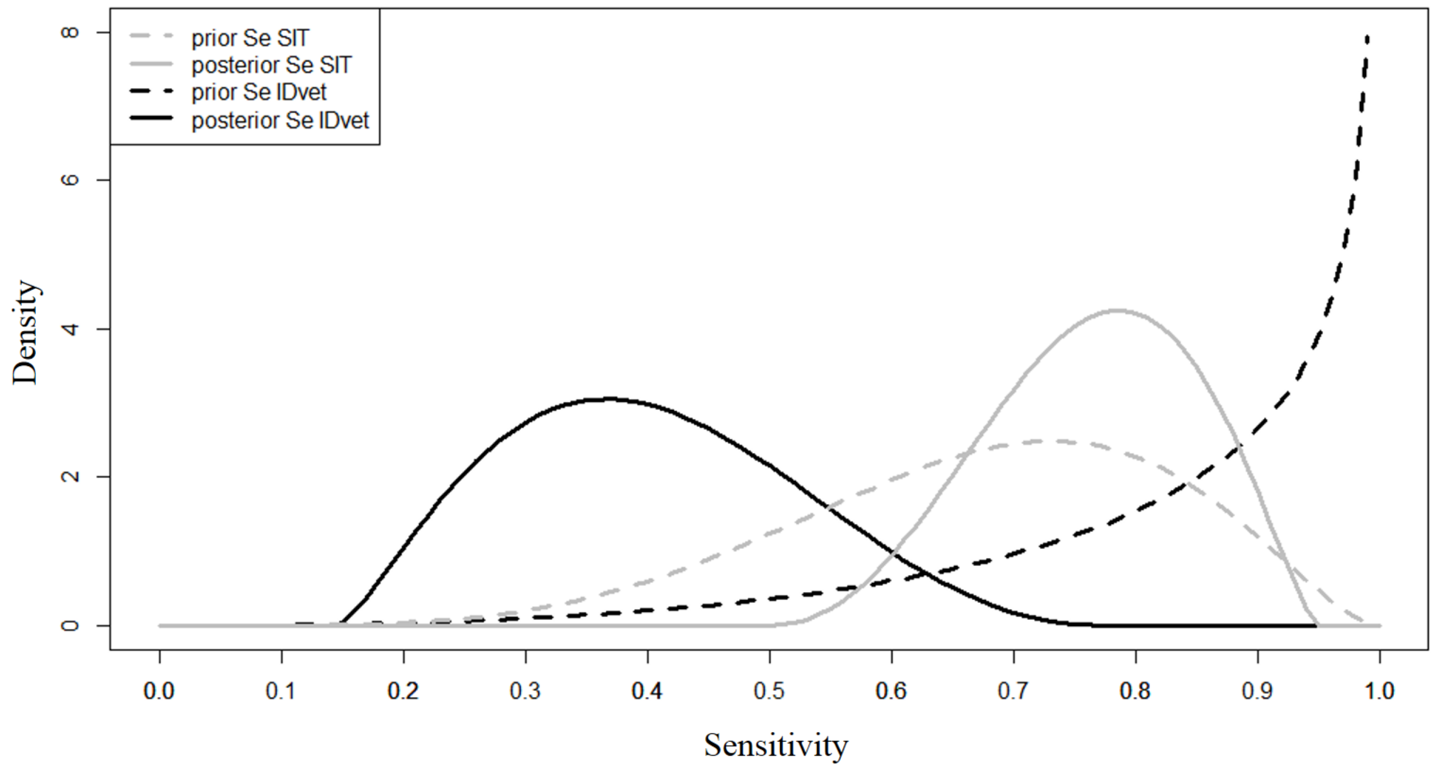

Supplement: Supplementary file 4 [file Image_1.PDF]
