# Supplementary material for: Evaluation of the Performance of the IDvet IFN-Gamma Test for Diagnosis of Bovine Tuberculosis in Spain
Source: Front Vet Sci. 2018 Sep 27;5:229. doi: 10.3389/fvets.2018.00229 (PMC6171474; doi:10.3389/fvets.2018.00229)

Supplementary Figure 2. Visual inspection of the Markov chains (SeSIT= Sensitivity of the SIT test; SpSIT= Specificity of the SIT test; SeIDvet= Sensitivity of the IDvet test; SpIDvet= Specificity of the IDvet test; piCAM= prevalence in the Region of Madrid; piCyL= prevalence in the region of Castilla y Leon).

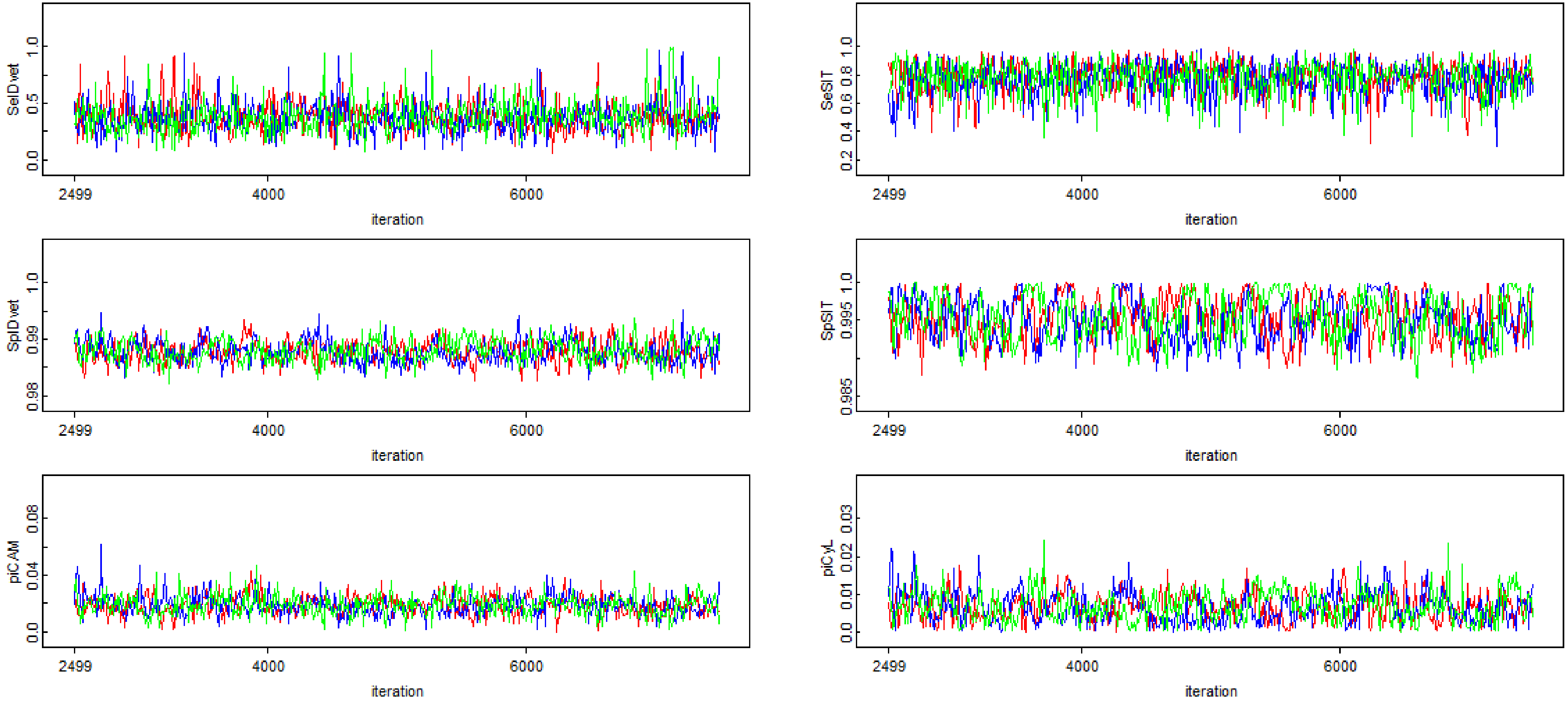

Supplement: Supplementary file 5 [file Image_2.PDF]
